# The complete mitochondrial genome of the bazaar fly, *Musca sorbens* Wiedemann (Diptera: Muscidae)

**DOI:** 10.1080/23802359.2018.1450677

**Published:** 2018-04-01

**Authors:** Ting Ma, Jia Huang

**Affiliations:** a Experimental Center, Guangdong Police College, Guangzhou, China;; b Department of Entomology, South China Agricultural University, Guangzhou, China;; c Department of Entomology, Cornell University, Ithaca, NY, USA

**Keywords:** Musca sorbens, bazaar fly, mitochondrial genome, phylogenetic analysis, Muscidae

## Abstract

The bazaar fly, *Musca sorbens* (Diptera: Muscidae) Wiedemann, 1830 is a world-wide species with sanitary, medical, and veterinary importance. The complete mitochondrial genome of *M. sorbens* is sequenced to better understand the mitogenomic characteristics and phylogeny of this species. The circular mitogenome is 16,120 bp in length, contains 13 protein-coding genes (PCGs), 22 tRNA genes (tRNAs), two rRNA genes, and an AT-rich control region. The mitogenome comprises an A + T content of 77.4%. All PCGs start with “ATN” codons except for *COI* which starts with TCG, and terminate with the common stop codons TNN. A phylogenetic tree, including six Muscidae species, is reconstructed based on the whole mitogenome sequences. The interspecific distances of mitogenomes between the six Muscidae species range from 0.059 to 0.168.

*Musca sorbens* Wiedemann, 1830, also known as bazaar fly, belongs to Muscidae, Diptera. It is a sanitary pest that distributes all over the world, with medical and veterinary importance. Up to the present, there are 561 species belonging to this large genus (retrieved from GBIF.org 2018), including one of the most medically important species, *Musca domestica* Linnaeus, 1758. Viewing it as a potential marker like *M*. *domestica* in medical entomology, the complete mitochondrial genome sequence of *M*. *sorbens* was present here for species identification and phylogenetic analysis (GenBank accession No. MG941012).

The specimens were collected from a snake carcass in Cangyuan, Lincang, Yunnan, China (23°54′28″N, 99°13′52″E) in May 2016. All of these specimens are deposited in Department of Entomology, South China Agricultural University, Guangzhou, China (SCAU). Twenty-six overlapping fragments were amplified using total genomic DNA as templates following the study of Zhang et al. (2013, 2015), except for the 25th primer pair was designed as 5′-AGGGTATCTAATCCTAGTT-3′ and 5′-TATAAATGGGGTATGAGCCC-3′ using available mitogenome sequences of Muscidae (Li et al. 2014; Lan et al. 2015). The amplified products were sequenced using ABI 3730xl DNA analyzer, after that assembled into a circular genome in MEGA 7.0.26 (Kumar et al. 2016).

The complete mitochondrial genome of *M*. *sorbens* was 16,120 bp in length, containing typical 37 genes (13 PCGs, protein-coding genes, 22 tRNA genes, and two rRNA genes) and a non-coding AT-rich control region as in other insects. Its arrangement was identical to the *M*. *domestica* mitogenome. The mitogenome of *M*. *sorbens* showed a high A + T biased (77.4%): with a base composition of A (39.1%), T (38.3%), G (9.5%), and C (13.1%). Higher A + T content of *M*. *sorbens* mtDNA was observed in the control region (86.0%). The length of tRNA genes ranged from 63 to 72 bp. All PCGs started with “ATN” codons except for *COI* which started with TCG, and terminate with the common stop codons TNN, which is similar to the previous results in other Diptera species (Chen et al. 2016; Ren et al. 2016; Zhu et al. 2016). The *lrRNA* and *srRNA* genes are 1364 and 786 bp in length, respectively.

Including one outgroup taxon in Drosophilidae (*Drosophila melanogaster*), a phylogenetic tree of complete mitogenomes of *M*. *sorbens* and all the other available Muscidae species with Refseqs in NCBI was established using neighbour-joining (NJ) method in MEGA. In general, all the six Muscidae species were clustered but weakly supported (Bootstrap, BP lower than 50) in the NJ tree (Figure 1); the outgroup taxon was clearly separated from the mitotypes of Muscidae; the three *Muscina* species were clustered with well supported (BP = 100). As expected, the mitogenome sequence of *M*. *sorbens* showed a close relationship (BP = 100) with the one of *M*. *domestica*. The interspecific distances of mitogenomes between the six Muscidae species ranged from 0.059 to 0.168, with an average of 0.131, and a variability of 0.032.

**Figure 1.**
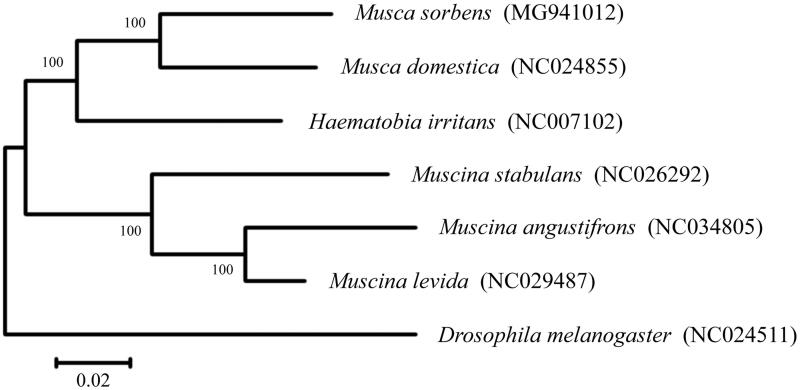
Neighbour-joining (NJ) tree reconstructed using six mitogenome sequences of Muscidae species and one outgroup taxon in Drosophilidae. The numbers above branches are Bootstrap (BP) values after re-sampling 1000 replicates. The bar indicates the estimated number of substitutions per site.
